# Supplementary material for: The Ability of the Hopkins Symptom Checklist-5 to Identify Generalized Anxiety Disorder and Major Depressive Disorder in the General Population
Source: Int J Environ Res Public Health. 2025 Apr 28;22(5):698. doi: 10.3390/ijerph22050698 (PMC12111364; doi:10.3390/ijerph22050698)
Supplement: Supplementary file 1 [file ijerph-22-00698-s001.zip › ijerph-3376936-supplementary.pdf]

Supplementary material

Table S1. Sensitivity, specificity, positive- and negative predictive value for all cut-off levels for Generalized anxiety disorder (GAD) or Major Depressive Disorder (MDD)

		CIDI-diagnoses		
		**GAD or MDD**	**GAD**	**MDD**
HSCL cut-off ≥ 1.20	Sensitivity	0.92	0.94	0.87
	Specificity	0.49	0.49	0.48
	PPV	0.07	0.05	0.04
	NPV	0.99	0.99	0.99
HSCL cut-off ≥ 1.40	Sensitivity	0.93	0.94	0.87
	Specificity	0.66	0.66	0.65
	PPV	0.11	0.07	0.05
	NPV	0.99	0.99	0.99
HSCL cut-off ≥ 1.60	Sensitivity	0.86	0.89	0.81
	Specificity	0.78	0.77	0.76
	PPV	0.14	0.09	0.07
	NPV	0.99	0.99	0.99
HSCL cut-off ≥ 1.75	Sensitivity	0.82	0.83	0.77
	Specificity	0.86	0.85	0.85
	PPV	0.21	0.13	0.10
	NPV	0.99	0.99	0.99
HSCL cut-off ≥ 1.80	Sensitivity	0.82	0.83	0.77
	Specificity	0.86	0.85	0.85
	PPV	0.21	0.13	0.10
	NPV	0.99	0.99	0.99
HSCL cut-off ≥ 2.00	Sensitivity	0.77	0.78	0.74
	Specificity	0.92	0.91	0.90
	PPV	0.29	0.19	0.16
	NPV	0.98	0.99	0.99
HSCL cut-off ≥ 2.20	Sensitivity	0.59	0.61	0.55
	Specificity	0.95	0.95	0.94
	PPV	0.36	0.24	0.19
	NPV	0.98	0.98	0.99
HSCL cut-off ≥ 2.25	Sensitivity	0.43	0.44	0.38
	Specificity	0.97	0.97	0.96
	PPV	0.41	0.27	0.20
	NPV	0.97	0.98	0.98
HSCL cut-off ≥ 2.40	Sensitivity	0.43	0.44	0.39
	Specificity	0.97	0.97	0.96
	PPV	0.41	0.28	0.21

	NPV	0.98	0.98	0.98
HSCL cut-off ≥ 2.60	Sensitivity	0.34	0.36	0.32
	Specificity	0.98	0.98	0.98
	PPV	0.51	0.35	0.27
	NPV	0.97	0.98	0.98
HSCL cut-off ≥ 2.80	Sensitivity	0.27	0.25	0.26
	Specificity	0.99	0.98	0.99
	PPV	0.56	0.33	0.30
	NPV	0.97	0.98	0.98
HSCL cut-off ≥ 3	Sensitivity	0.23	0.19	0.26
	Specificity	0.99	0.99	0.99
	PPV	0.59	0.32	0.36
	NPV	0.97	0.98	0.98

Hopkins Symptom Checklist-5 (HSCL)

PPV= positive predictive value, NPV= negative predictive value.

Table S2. Sensitivity and specificity of Hopkins Symptom Checklist-5 at different cut-off levels for different CIDI-diagnoses (30days)

		**Any affective or anxiety disorder***	**Any anxiety disorder**	**Any affective disorder**
HSCL cut-off ≥ 1.80	Sensitivity	0.45	0.42	0.69
	Specificity	0.88	0.86	0.85
	PPV	0.35	0.29	0.11
	NPV	0.92	0.92	0.99
HSCL cut-off ≥ 2.00	Sensitivity	0.36	0.33	0.66
	Specificity	0.92	0.92	0.90
	PPV	0.43	0.35	0.16
	NPV	0.91	0.92	0.98
HSCL cut-off ≥ 2.25	Sensitivity	0.19	0.18	0.36
	Specificity	0.98	0.97	0.96
	PPV	0.54	0.47	0.22
	NPV	0.89	0.90	0.98

Hopkins Symptom Checklist-5 (HSCL)

* Including generalized anxiety disorder, panic disorder, specific phobia, agoraphobia, social anxiety disorder, major depressive disorder, bipolar type I and II disorders

Table S3. Sensitivity and specificity for different cut-off levels for Generalized anxiety disorder (GAD) or Major Depressive Disorder (MDD), for women and for men

			**GAD or MDD**
Women, n= 860	HSCL cut-off ≥ 1.80	Sensitivity	0.85
		Specificity	0.84
	HSCL cut-off ≥ 2.00	Sensitivity	0.78
		Specificity	0.91
	HSCL cut-off ≥ 2.25	Sensitivity	0.40
		Specificity	0.97
Men, n= 483	HSCL cut-off ≥ 1.80	Sensitivity	0.73
		Specificity	0.89
	HSCL cut-off ≥ 2.00	Sensitivity	0.73
		Specificity	0.93
	HSCL cut-off ≥ 2.25	Sensitivity	0.55
		Specificity	0.97

Table S4. Sensitivity and specificity for different cut-off levels for Generalized anxiety disorder (GAD) or Major Depressive Disorder (MDD), for participants below and above 30 years.

			**GAD or MDD**
Participants ≤ 30 years, n= 434	HSCL cut-off ≥ 1.80	Sensitivity	0.87
		Specificity	0.81
		PPV	0.26
		NPV	0.99
	HSCL cut-off ≥ 2.00	Sensitivity	0.83
		Specificity	0.88
		PPV	0.34
		NPV	0.98
	HSCL cut-off ≥ 2.25	Sensitivity	0.43
		Specificity	0.96
		PPV	0.45
		NPV	0.96
Participants >30 years, n= 909	HSCL cut-off ≥ 1.60	Sensitivity	0.85
		Specificity	0.80
		PPV	0.11
		NPV	0.99
	HSCL cut-off ≥ 1.80	Sensitivity	0.77
		Specificity	0.88
		PPV	0.16
		NPV	0.99
	HSCL cut-off ≥ 2.00	Sensitivity	0.69
		Specificity	0.94
		PPV	0.25
		NPV	0.99
	HSCL cut-off ≥ 2.25	Sensitivity	0.42
		Specificity	0.97
		PPV	0.37
		NPV	0.98
